# Bio-Ecology of the Louse, *Upupicola upupae*, Infesting the Common Hoopoe, *Upupa epops*

**DOI:** 10.1673/031.011.4601

**Published:** 2011-04-11

**Authors:** G. P Agarwal, Aftab Ahmad, Archna Rashmi, Gaurav Arya, Nayanci Bansal, A.K. Saxena

**Affiliations:** Department of Zoology, Government Raza Postgraduate College, Rampur (U.P.) 244901, India

**Keywords:** Ischnocera, Phthiraptera, *Upupicola upupae*

## Abstract

The population characteristics of the louse, *Upupicola upupae* (Shrank) (Mallophaga: Philopteridae: Ishnocera), infesting the Common Hoopae, *Upupa epops* L. (Aves: Upupiformes), were recorded during 2007–08 in District Rampur, Uttar Pradesh India. The pattern of frequency distribution of the louse conformed to the negative binomial model. The lice and its nits were reared *in vitro* at 35 ± 1° C, 75–82 % RH, on a feather diet. The data obtained was used to construct the life table and to determine the intrinsic rate of natural increase (0.035 female/day), the net reproductive rate was 3.67 female eggs/female, the generation time was 37 days, and the doubling time of the population was 19 days. The chaetotaxy of the three nymphal instars has also been noted to record their diagnostic characteristics. Information on egg morphology and antennal sensilla is also presented.

## Introduction

The population characteristics of Phthiraptera on selected Indian birds have been recorded from time to time (Chandra et al. 1990; Singh et al. 1991; Trivedi et al. 1992; Saxena et al. 1995; Gupta et al. 2007; Beg et al. 2008; Khan et al. 2008; Rajput et al. 2009). Studies on the *in vitro* bionomics of avian lice have been performed by Wilson (1934, 39), Arora & Chopra (1959), Agarwal (1967), Williams (1970), Saxena et al. 1995, and Singh et al. 2001. In case of certain avian Ischnocera the data obtained from *in vitro* experimentation have been utilized to construct life tables to determine the intrinsic rate of natural increase (Beg et al. 2005; Saxena et al. 2007; Gupta et al. 2007; Saxena et al. 2009). Survey of literature revealed that bio-ecological studies on phthirapterans occurring on the Common Hoopae, *Upupicola epops* L. (Aves: Upupiformes: Upupidae), deserved investigations.

The present report supplements information on the population characterstics, *in vitro* bionomics, life table statistics, egg morphology, and chaetotaxy of three nymphal instars of an ischnoceran louse, *Upupicola upupae* (Shrank) (Mallophaga: Philopteridae: Ischnocera) infesting *U. epops.*

## Materials and Methods

Thirty *U. epops* were live trapped in the district Rampur (28° 49′ 12″ N 79° 1′ 11″ E, in Uttar Pradesh state of India) during 2007–08 and subjected to delousing, at first manually and then by fumigation (Saxena et al. 2007). The examined birds were released in the wild in district Rampur after delousing. The louse load was placed in 70% alcohol and separated according to sex and stage. The population parameters (prevalence, mean, sample mean abundance, variance, exponent of negative binomial, and index of discrepancy) were computed (Rozsa et al. 2000). The quality of fit between the observed and expected frequencies was determined by χ^2^. The egg laying sites were recorded by visual inspection. Feathers bearing eggs were gently cut and subjected to scanning electron microscopy. Freshly laid eggs and adults were reared *in vitro* condition (35 ± 1° C, 75–82 % RH, feather diet) to record the bionomics on the lines adopted by Gupta et al. (2007) and Saxena et al. (2007). The data obtained from *in vitro* experimentation was used to construct the life table and to compute the intrinsic rate of natural increase. Different instars of nymphs were examined using light microscopy for recording the chaetotaxy. The adults were also examined using SEM.

## Results

### Population characteristics

Only one phthirapteran species *U. upupae* was recovered from 30 Common Hoopae in the district Rampur during 2007–08. Its prevalence was 40% (n=30). A total of 468 specimens were recovered (mean intensity-39.0; sample mean abundance- 15.6; range of infestation 7–83). The male female ratio of adults was 1:1.3. The adult nymph ratio was 1: 1.4. The ratio of three nymphal instars was 1:0.8:0.7.

The pattern of frequency distribution was skewed (variance to mean ratio — 39.33). The value of the exponent of the negative binomial (k) and the index of discrepancy (D) were 0.12 and 0.715, respectively. The pattern of frequency distribution of *U. upupae* conformed to the negative binomial model (χ^2^ = 13.21, P > 0.05).

**Table 1.**
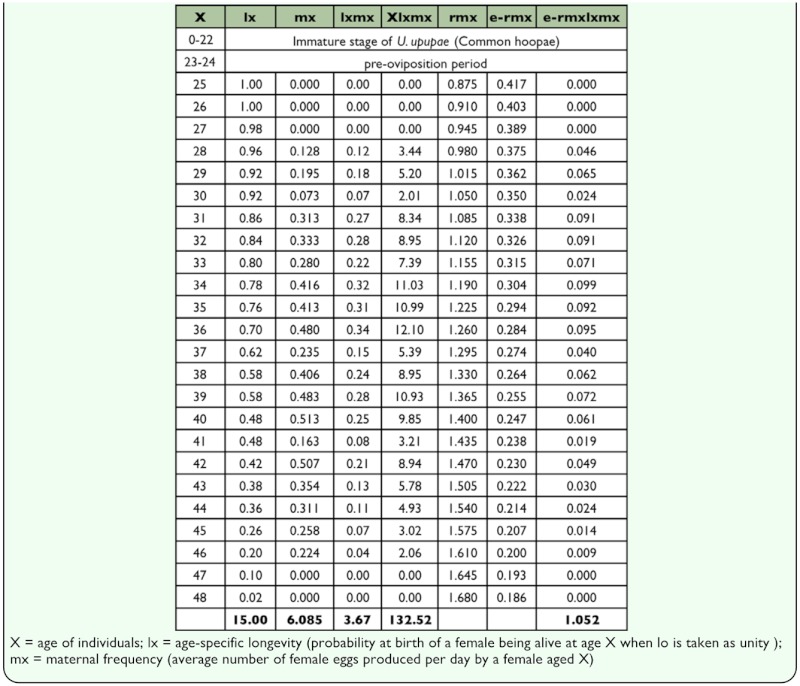
Lifetable, fecundity and rate of natural increase of Commom Hoopae louse (*Upupicola upupae*).

***In vitro* bionomics and life table statistics** The adults of *U. upupae* inhabit the wings and crest feathers of *U. epops.* The nymphs usually occupy the basal part of neck feathers. Most of the eggs of this louse occurred on the wings and crest feathers (Figure 4A).

The incubation period of the eggs was 5.25 ± 0.97 days (range 4–7 days, n = 88). The duration of three nymphal instars was 5.08 ± 0.85 days (range 4–7 days, n = 74), 5.54 ± 0.84 days (range 4–7 days, n = 55), and 6.04 ± 1.89 days (range 5–7 days, n = 25). The lifespan of adult females (15.0 ± 6.28 days, range 2–24 days, n = 50) was longer than that of males (10.96 ± 3.92 days, range 1–21 days, n = 50). An adult female laid an average of 6.1 eggs during its lifespan, at a rate 0.35 egg/f/day (*in vitro* condition; 35 ±_1° C, 75– 82% RH, feather diet). These data were utilized to construct the life table of the louse after making certain assumptions. The intrinsic rate of natural increase (rm) was determined as 0.035/day (Table 1) by the equation Σe^-rmxlxmx^=1 (where e is the base of natural logarithm, x is the age of individuals in days, 1x is the number individual alive at age x, and mx is the number of female offsprings produced/female) (Howe 1953). The net reproductive rate (Ro=1xmx) was 3.67 female eggs/female. The generation time (T= log e Ro/rm) was found to be 37.15 days. The doubling time of population (DT= log2/logλ) was ascertained as 19.1 days.

**Figure 1.**
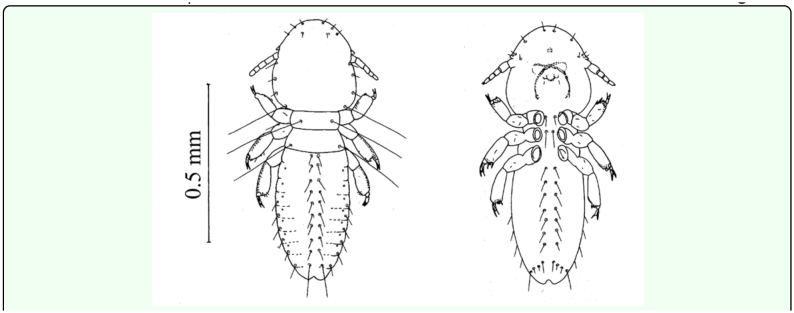
Dorsal and ventral views of 1^st^ instar *Upupicola upupae.* High quality figures are available online.

### Nymph Morphology

**First instar: (0.8- 0.9 mm) (Figure 1).** The head is longer than it is broad, the anterior margin is round, and a faint pre-antennal suture extends across the head; the post-antennal region is widest immediately behind eyes. Only the cutting edges of the mandibles show slight pigmentation. The hypopharynx and gular plate are not distinct. The antennae are filiform and five segmented in which the fifth segment is shortest and the second segment is broadest. The prothorax is rectangular and on the pronotum a long seta is present on each side. The pterothorax is larger than the prothorax and its posterior margin is slightly concave and bears one long setae on each postero-lateral angle. The first pair of legs is shorter than the others. The claws of meso- and meta-thoracic legs are longer and more slender than those of the pro-thoracic legs. Each leg is provided with a number of setae.

The abdomen is more or less cylindrical without any pigmentation (Figure 1). It consists of ten recognizable segments of which the apparent first is interpreted_as segment II. The abdomen is broadest at segment V–VI, and segment IX–X and XI segment taper posteriorly. Segment II is slightly longer than the others and segment XI is the shortest and its posterior margin is emarginated. In the first instar segments IX and X are not fused, unlike the adult. A pair of spiracles is present on segment III–VIII. The tergal, sternal, and pleural chaetotaxy and measurements of the first instar nymph are given in Table 2.

**Figure 2.**
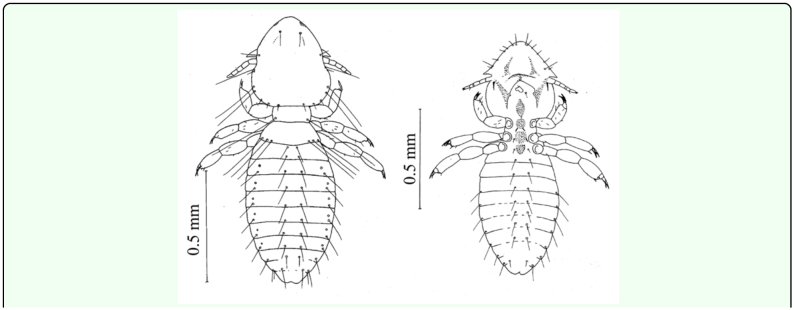
Dorsal and ventral views of 2^nd^ instar *Upupicola upupae.* High quality figures are available online.

**Second instar nymph: (1.18- 1.23 mm) (Figure 2).** The second instar differs from the first instar in shape, size pigmentation, and chaetotaxy. The head is not as rounded anteriorly as in the previous instar and the narrow pre-antennal suture has become more distinct (Figure 2). Coni are also better developed. Mandibles are pigmented; the hypopharynx is delineable and the gular plate is also differentiated. In the prothorax there is very little change in shape; it however, shows pigmentation of the pronotum and the seta has grown longer and shifted nearer the postero-latertal angle on each side. In the pterothorax the posterior margin shows greater concavity, and pteronotum is fully pigmented. Two setae are added each side to the already existing seta; these seta are long and extend up to the posterior margin of segment IV. Ventrally pro-, meso-, and meta-sternal plates are distinct and two setae are present on each external plate. Legs are well developed and slightly pigmented on the dorsal side of the femur and tibia. The abdomen showed an increase in length and breadth (Figure 2). Lateral pigmented areas have appeared on segment II–IV. Both segmentation and chaetotaxy of the abdomen almost remain as in the previous instars; there is, however, slight difference in the number of pleural setae.

**Table 2.**
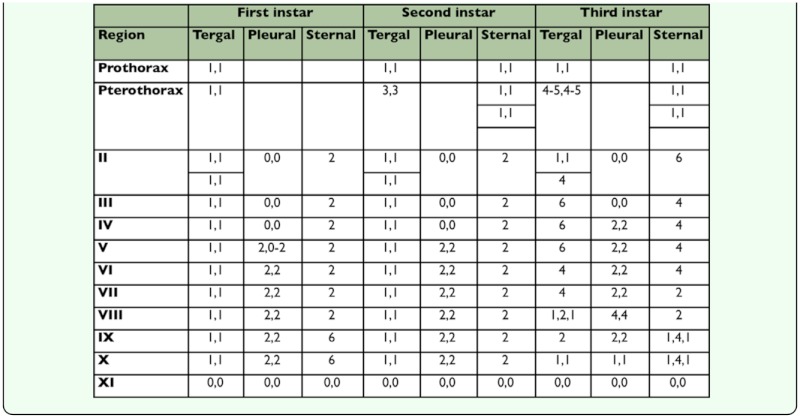
Showing chaetotaxy of first, second and third instar of *Upupicola upupae.*

**Figure 3.**
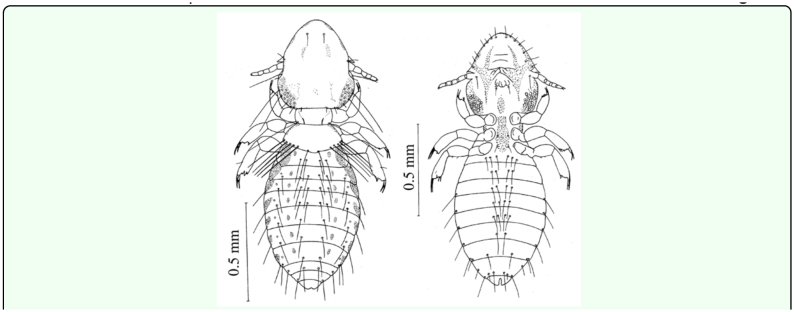
Dorsal and ventral views of 3^rd^ instar *Upupicola upupae.* High quality figures are available online.

**Third instar nymph: (1.39–1.42) (Figure 3).** The third instar nymph resembles the second instar nymph. Pigmentation on head and thorax becomes darker and the pigmented areas on the abdomen have increased and so have the number of setae. The head is slightly more pointed anteriorly than the second instar nymph and the coni is now prominent (Figure 3). The hypopharynx and gular plate are further differentiated. The thorax is similar in shape to that of the second instars nymph, but it is further pigmented (Figure 3). The prothorax bears two setae, one on each postero-lateral angle. In the pterothorax 1–2 setae are added and now 5 setae are present on the posterior margin of pterothorax (on each side).

The abdomen shows increase in pigmentation (Figure 3). Lateral pigmented areas representing the pleurites are present from segment II–VIII. Besides these, there are oval pigmented areas in segments II–VIII, one on each side. There is no change in segmentation. However, there is marked increase in the number of tergal, pleural, and sternal setae (Table 2).

**Figure 4.**
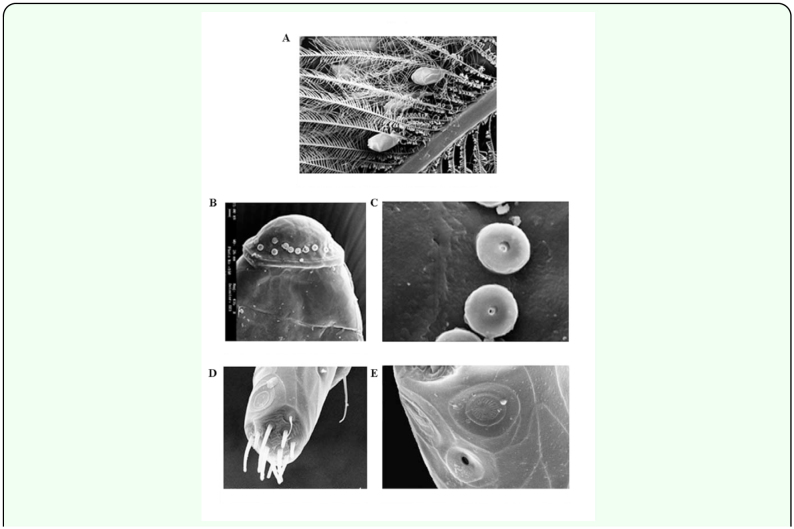
(A) Photograph showing egg laying pattern of *Upupicola upupae.* (B) Scanning electron microscope photograph of the anterior portion of the egg of *Upupicola upupae.* (C) Enlarged view of Figure B, showing detailed nature of micropyles. (D) SEM photograph of the third flagellomere of *Upupicola upupae,* showing the sensilla present on the apical end and antero-lateral surface. (E) More enlarged view of Figure D to show the nature of placodean sensilla and pit organ.. High quality figures are available online.

**Egg morphology.** The egg shell of *U. upupae* is a miniature rice grain like structure. The egg chorion does not show any sculpturing or projections. The dome shaped opercular disc is also smooth in nature and bears 28–30 button shaped micropyles, which are not arranged in single line (irregularly placed), along the rim of operculum (Figure 4B, C). The stigma remains concealed in the cementing material.

**Antennal sensilla.** The second flagellomere of antenna bears two distinct placodean sensilla (saucer shaped structure having central groove with numerous radiating ridges separated by grooves) and a pit sensilla (coelomic sensilla). The apical end of second flagellomere bears 10 tactile sensilla (seta and pegs) of varying length (Figure 4D, E).

## Discussion

Two phthirapteran species reportedly infest the *U. epops* (Price et al. 2003). During the present study only one ischnoceran louse (*U. upupae*) was recovered. The presence of the amblyceran louse, *Menacanthus fertilis* was not recorded. The frequency distribution pattern of *U. upupae* on *U. epops* was aggregated and conformed to the negative binomial model. Rekasi et al. (1997) computed that frequency distribution pattern of 21 (out of 27) species occurring on 13 birds conformed to negative binomial model. On the other hand Saxena et al. (2007), Gupta et al. (2007), Beg et al. (2008), and Rajput et al. (2009) found that negative binomial distribution was found to be a good fit in case of only one louse (out of 19). The sex ratio of *U. upupae* was female biased, like most of the other phthirapterans (Marshall 1981). Reasons responsible for skewed sex ratio have been discussed by Gupta et al. (2007).

The incubation period of eggs, duration of three nymphal stages and the adult lifespan of *U. upupae* does not show any departure from the other ischnoceran species studied, so far (Wilson 1934, 1939; Arora and Chopra, 1959; Agarwal 1967; Williams 1970; Saxena et al. 1991; Singh et al. 2001; Gupta et al. 2007; Saxena et al. 2007, 09). The rate of egg production of different species recorded by the aforesaid workers varied from 0.4 to 2.0 eggs/female/day. In case of eight avian lice the data obtained from *in vitro* experimentation have been utilized to construct a life table and to determine the intrinsic rate of natural increase, as well as the doubling time of the population (Beg et al. 2005; Saxena et al. 2007; Gupta et al. 2007; Saxena et al. 2009). The intrinsic rate of natural increase of different species determined by the aforesaid workers varied from 0.033 to 0.074. Thus, *U. upupae* (rm= 0.037) appears to be a slow breeder in this context. The present report provides further information on the distribution of *U. upupae* on host bird, the egg laying sites, and the egg morphology. The eggshell of *U. upupae* differs from that of *Degeeriella regalis* (V. Khan, Govt. Raza P. G. College, Rampur, personal communication) as the operculum of the latter bears hexagonal ridges and only 14–16 micropyles.

Specific studies on the morphology of nymphal instars have been made by Modrejeweska and Zlotorzycka (1987) and Mey (1994). Price and Hellenthal (1986) have highlighted the importance of nymph morphology for determining the louse relationship. Smith (2000) has further noted the ontogenic transformation in shape of head of three instars of nymph of four avian lice and recommended the use of nymph morphology to establish the phylogeny of bird lice. The present study provides the diagnostic features of three instars of nymphs of *U. upupae.* Three instars of nymphs of *U. upupae* differ not only in size, segmentation, chitinization, but also in gradual accumulation/displacement of setae on different parts of body. In adult females of *U. upupae* the three segments (IX, X, XI) become fused.
